# Regulation of progesterone during follicular development by FSH and LH in sheep

**DOI:** 10.1590/1984-3143-AR2022-0027

**Published:** 2022-07-11

**Authors:** Ziqiang Ding, Hongwei Duan, Wenbo Ge, Jianshu Lv, Jianlin Zeng, Wenjuan Wang, Tian Niu, Junjie Hu, Yong Zhang, Xingxu Zhao

**Affiliations:** 1 College of Veterinary Medicine, Gansu Agricultural University, Lanzhou, China; 2 Gansu Key Laboratory of Animal Generational Physiology and Reproductive Regulation, Lanzhou, China; 3 Key Lab of New Animal Drug Project of Gansu Province, Key Lab of Veterinary Pharmaceutical Development of Ministry of Agriculture and Rural Affairs, Lanzhou Institute of Husbandry and Pharmaceutical Sciences of CAAS, Lanzhou, China

**Keywords:** follicle-stimulating hormone, granulosa cells, luteinizing hormone, progesterone, sheep

## Abstract

Progesterone (P4) can participate in the development of female mammalian antral follicles through nuclear receptor (PGR). In this experiment, the differences of P4 synthesis and PGR expression in different developmental stages of sheep antral follicles (large > 5mm, medium 2-5mm, small < 2mm) were detected by enzyme-linked immunosorbent assay, immunohistochemistry, qRT-PCR and Western blotting. Secondly, sheep follicular granulosa cells were cultured in vitro. The effects of different concentrations of FSH and LH on P4 synthesis and PGR expression were studied. The results showed that acute steroid regulatory protein (StAR), cholesterol side chain lyase (P450scc) and 3β Hydroxysteroid dehydrogenase (3β-HSD) and PGR were expressed in antral follicles, and with the development of antral follicles in sheep, StAR, P450scc and the expression of 3β-HSD and PGR increased significantly. In vitro experiments showed that FSH and LH alone or together treatment could regulate P4 secretion and PGR expression in sheep follicular granulosa cells to varying degrees, hint P4 and PGR by FSH and LH, and LH was the main factor. Our results supplement the effects of FSH and LH on the regulation of P4 synthesis during follicular development, which provides new data for further study of steroid synthesis and function in follicular development.

## Introduction

Follicle is an important tissue structure of female reproductive development, which is composed of oocytes, granulosa cells and theca cells (Barnett et al., 2006). The hormone secretion profile (quantity, type of hormone) changes along the follicle development such as granulosa cells (GCs) and theca cells change, resulting in the change of various steroid hormones in follicles, so as to regulate the reproductive physiological process of female animals (Holesh et al., 2021; Duffy, 2015). Follicular granulosa cells are important models to study the regulation mechanism of steroid hormone secretion in vitro. Granulosa cells can secrete many kinds of steroid hormones, which play an important role in the growth and development of follicles (Drummond, 2006), and progesterone (P4) is one of them.

P4 is produced by blood cholesterol catalyzed by acute steroid regulatory protein (StAR), cholesterol side-chain lyase (P450scc), and 3β hydroxyl steroid dehydrogenase (3β-HSD) (Sekar et al., 2000a), and is an important steroid hormone for the maintenance of reproductive physiological processes in animals. Expression of P4 synthetase has been found in human, bovine and mice ovaries, indicating that the ovary has the ability to synthesize P4 (Erickson et al., 1991; Garverick et al., 2002; Ji et al., 2020). The P4 content was lowest in GCs of immature follicles, and P4 synthesis in GCs was significantly increased after treatment with human chorionic gonadotropin (hCG) (Natraj and Richards, 1993). Study have shown that relatively low concentrations of P4 in the blood can prevent follicles from developing properly, and the dominant follicles cannot ovulate normally to become persistent follicles (Bartlewski et al., 2017). LH secretion increases P4 levels, P4 can promote the synthesis of proteolytic enzymes and induce the ovulation of follicles (Sirois and Fortune, 1990; Stock and Fortune, 1993). which was also demonstrated by prolonged follicular development in rats treated with the P4 antagonist RU486 (Iwamasa et al., 1992). The above studies have proved that P4 is involved in follicular development in sheep and rats.

P4 regulates the expression of genes involved in ovulation and/or luteinization via its specific nuclear receptor, PGR (Mishra et al., 2015). PGR has been localized in GCs of mice, pigs, bovines, macaques and humans (Sriraman et al., 2010; Durlej et al., 2010; D'Haeseleer et al., 2007; Bishop et al., 2016; Sueldo et al., 2015). Furthermore, in rabbits and pigs, it was found that PGR expression in utero has effects on implantation, survival, and development of early embryos (Peiró et al., 2008; Chen et al., 2016). PGR also plays an important role of LH induced follicular rupture (Sriraman et al., 2010). In PGR knockout mice, ovulatory follicles developed but failed to ovulate (Lydon et al., 1995). In genomic studies, PGR has been shown to be an essential gene for the successful release of an oocyte from an ovulatory follicle (Akison and Robker, 2012). Furthermore, PGR can promote anti-apoptotic effects and maintain cell survival during ovulation (Hughes and Murphy, 2021).

FSH and LH are important reproductive hormones that play different biological roles in follicular development (Gong et al., 1996). They are secreted by the pituitary gland. By binding to their respective specific receptors, they activate cyclic adenosine monophosphate (cAMP)-dependent physiological processes, thereby regulating steroid-related synthetase activity in GCs (Sekar et al., 2000b; Shih et al., 2011). FSH induces the expression of cytochrome P450 aromatase (CYP19) and the secretion of estradiol (E2) in GCs (Nakano et al., 2020), and regulates the synthesis of dihydrotestosterone (DHT) and the expression of androgen receptor (AR) in GCs with antral follicles, thereby regulating GC function (Duan et al., 2021). LH regulates the ovulation-related ERK1/2 pathway by inducing growth factor activation (Herbison, 2016), and is also involved in the regulation of androgen synthesis in granulocytes (Pizzo et al., 2015). In serum-free luteinized GCs cultured in minimum essential media (MEM), LH alone promoted the synthesis of progesterone and cyclic adenosine phosphate (Sekar et al., 2000b). The levels of FSH and LH change dynamically with follicular development. Therefore, it is important to explore the regulation of P4 by FSH and LH in preovulatory follicles.

Therefore, we used sheep follicles as an experimental model to detect the expression and localization of P4 synthsis and its receptor in ovarian tissue, as well as the expression in large, medium, and small follicles. GCs were treated in vitro with FSH and LH to detect the effects of different concentrations of FSH and LH on the expression of P4 synthsis and receptor, and to provide supplementary information for further studies of steroid synthesis in sheep ovarian GCs.

## Methods

### Animals and tissue collection

All the experimental procedures were performed according to the rules of the Gansu Agricultural University Animal Ethics and Guidelines (approval number: GSAU-AEW-2017-0003). Sheep (small-tailed Han sheep, aged about 1 year and weighing about 25-35 kg). Normal ovaries on both sides of the uterus (no luteum and lesions were observed, n=80) were selected and placed into phosphate buffer normal saline (PBS) prepared at 30-37 °C containing 100 mg/ mL streptomycin and 100 U/ml penicillin, and transported back to the laboratory within 30 min. According to the gonadotropin-dependent characteristics of follicles, follicles were divided into large (diameter > 5 mm), medium (diameter 2-5 mm), and small (diameter < 2 mm) types. 65 of the 80 ovaries were randomly selected and used for follicular fluid extraction from large (n = 31) medium (n = 44) small (n = 96) follicles with the use of sterile syringe (26G, 0.45mm x10mm) and was centrifuged at 1500 g for 5 min, collect deposits. The supernatant was filtered through a 0.45 μm filter for enzyme-linked immunosorbent assay (ELISA). The GCs extracted from ovaries were deposited and stored in a refrigerator at -80 °C for quantitative reverse transcription polymerase chain reaction (qRT-PCR) and Western blot analyses. 10 normal ovaries not extracted were used in cell culture, and another 5 ovaries were fixed with 4% neutral paraformaldehyde (pH = 7.4) for immunohistochemistry.

### Culture and treatment of GCs

Fresh ovarian samples (n = 10) were washed three times with PBS which contained a antibody at 37 °C and then brought into laboratory. The follicular fluid sucked from follicles with the use of sterile syringe (1 mm) was placed in a 1.5 ml centrifuge tube, centrifuged at 2,000g for 5 min, and the supernatant was discarded. After washing three times, 1 g/L hyaluronidase was added for digestion for approximately 10 min, and the fluid was filtered into a new 1.5 mL centrifuge tube with a 0.074 mm filter. Dulbecco’s MEM (DMEM/F12, Solarbio, Beijing, China) containing 10% (v/v) fetal bovine serum (Hyclone, Logan, UT), penicillin (50 IU/mL), and streptomycin (50 µg/mL), preparing cell suspension, and inoculating into 25 ml cell culture bottles, and the cell culture medium was changed every 48 h. When the cells grew to 85–90%, they were washed with PBS preheated at 37 °C three times. Subsequently, 1 mL of 0.25% trypsin-ethylenediamineetetraacetic acid was added to digest the granulosa cells for approximately 60s. The cell suspension was centrifuged and subcultured in a six-well plate (Corning Company) which contained 2 mL of culture medium. The incubator conditions were: 37 °C and 95% O_2_/5% CO_2_. DMEM / F12 medium without FBS was added for 12 h before treatment.

In order to study effects of FSH and LH on P4 related proteins, we added different concentrations of FSH and LH (1 IU/mL, 0.1 IU/mL, 0.01 IU/mL, pituitary, sheep, Solarbio, Beijing, China) in GCs cultured in vitro (Duan et al., 2021). Expected concentration FSH and LH was added to serum-free DMEM / F12 medium for 24 h and the same volume of serum-free medium was added to the control group.

### Enzyme-linked immunosorbent assay (ELISA)

P4 secretion levels of follicles of different sizes and GCs after treatment were detected by ELISA (sheep P4 kit, Yingxin, Shanghai, China). According to the manufacturer's instructions, the absorbance of the sample and the blank well were measured at 450 nm within 15 min, the absorbance of the sample was subtracted from the absorbance of the blank well and the P4 level was expressed as ng/mL.

### Immunohistochemical

Tissue sections (thickness of 4 μm) were dewaxed and repaired, washed three times with PBS (pH = 7.4) for 5 min, and incubated with 3% hydrogen peroxide at 37 °C for 20 min to remove endogenous peroxidase. After washing with PBS, goat serum was used to block for 20 min at 37 °C to reduce nonspecific binding sites. This process was followed by incubation at 4 °C for ≥ 14 h (StAR 1:500 dilution, bs-3570R, P450scc 1:500 diluted, bs-10099R, PGR 1:500 diluted, bs-23376R. All the antibodies used herein were purchased from Bioss, Beijing, China) and PBS-treated negative control group without adding any target enzymes. After the end of incubation, biotin secondary antibody was used for labeling at 37 °C for 20 min, and horseradish peroxidase binding labeling site was used at 37 °C for 20 min, 3,3'-diaminobenzidine (DAB) was used for color development, hematoxylin was counterstain, hydrochloric acid and alcohol differentiation processes were performed, and clean in distilled water. The glass slide were dehydrated and sealed, and the staining results were observed and photographed with a light microscope (Olympus DP73, Olympus, Tokyo, Japan).

### Immunocytochemistry

PGR expression in sheep follicular GCs was detected using immunofluorescence. Cultured GCs were fixed in 4% (v/v) paraformaldehyde, washed with 4 °C PBS, treated with 0.1% (v/v) Triton X-100 for 30 min, and then incubated with 5% (w/v) bovine serum albumin (BSA) for 30 min. The cells were then mixed with polyclonal rabbit anti-PGR (1:300, bs-23376R, Bioss, Beijing, China) and with 2% BSA (negative control). Detection of PGR antibody with fluorescein isothiocyanate (FITC)-coupled goat anti-mouse immunoglobulin (IgG) (H+L) antibody (1:200, TransGen, Beijing, China), was then treated with 1 μg/ml 4,6 diamidino 2 phenyl indole (4’6’-diamindino-2-phenylindole, Solarbio, Beijing, China), and was used for nuclear reverse staining. Digital image acquisition was performed with an Olympus DP73 optical microscope (Olympus, Tokyo, Japan).

### Total ribonucleic acid (RNA) extraction and qRT-PCR

Samples of cells were stored at -80 °C. TRIzol (Solarbio, Beijing, China), total RNA was released following cell lysis. The absorbance at A260/280 nm was measured with the use of a micronucleic acid analyzer, and the purity and integrity of RNA were detected by electrophoresis. Reverse transcription was conducted with the use of the Prime Script RT reagent kit with a gDNA eraser (Takara, Dalian, China) to obtain complementary deoxyribonucleic acid (cDNA). Addition of 10 µl 2× SYBR Green II PCR mixture (TaKaRa), 25 mol/L forward primer, reverse primer, 2 µL template, and ddH_2_O, the total volume was determined to be 20 µL with the use of a lightcycler 480 real-time detection system (Roche, Basel, Switzerland). The transcription levels of *StAR*, *P450scc*, *3β-HSD*, and *PGR* in follicles and cultured GCs at different stages were evaluated by qPCR, with β-actin as the reference gene. The setting conditions were 95 °C for 10 s, 95 °C for 5 s, and 60 °C for 30 s. From 60 °C to 95 °C, the melting curve was obtained by increasing the temperature by 0.5 °C every 5 s. The primer sequences used are listed in Table 1. The result was calculated using2^-ΔΔCt^ (Livak and Schmittgen, 2001).

**Table 1:** Primers used for qRT‐PCR.

**Genes**	**Primer Sequences(5’-3’)**	**Length(bp)**	**Accession No.**
*StAR*	F: TGCCGAAGACCATCATCAA	111	NM_001009243.1
	R: GCCTTCAACACCTGGCTTC		
*3β-HSD*	F: GAAGCTAATGGGTGGGCTCT	141	NM_001135932.1
	R: ATTGGTCAGGATGCCGTTG		
*P450scc*	F: GGCTCCAGAGGCAATAAAGAAC	149	NM_001093789.1
	R: ACTCAAAGGCAAAGCGAAACA		
*PGR*	F: GTCAGGCTGGCATGGTTCTT	123	Z66555.1
	R: GGGCTTGGCTTTCATTTGG		
*β-Actin*	F: CCATTGAGCACGGCATTGT	184	U39357.2
	R: GCAGGGGTGTTGAAGGTCTC		

### Western blotting

Took out the preserved sample, added RIPA protein lysate and protease inhibitor PMSF, fully lysed, 12,000 × g, centrifuge at 4 °C for 15min. Next, collected the supernatant, extracted the total protein, and used BCA protein detection kit (BioTek) to detect the protein concentration. Then added the collected protein supernatant to 5 × Loading Buffer, fully mixed, denature at 98 °C for 15min and store at -20 °C. The Western blot technique (Xiao et al., 2018) developed by our laboratory was used to separate the protein by polyacrylamide gel electrophoresis (SDS-PAGE) and transferred to a polyvinylidene fluoride membrane (Millipore, Atlanta, GA, USA). The electrotransfer solution was washed away with 1× trimethylaminomethane buffer saline (TBST) and was sealed with TBST which contained 5% skimmed milk powder for 1 h. The antibody was incubated with rabbit polyclonal antibody StAR (bs-3570R, 1:500, Bioss) P450scc (bs-10099R, 1:500, Bioss) PGR (bs-23376R, 1:500, Bioss) with β-actin (bs-0061R, 1:3,000, Bioss) as an internal reference, overnight at 4 °C, after washing, goat anti-rabbit secondary antibody (bs-0295G-HRP, Bioss) at a dilution ratio of 1:3,000, at 37 °C for 1 h. Immune complexes were detected with enhanced chemiluminescence solution (Abnova, Taibei, Taiwan), and the expression was quantified using ImageJ (National Institutes of Health).

### Data analyses

SPSS (version 22.0, IBM Corporation, NY, USA) was used for statistical analyses, and all data are expressed as mean ± standard error of the mean (SEM). All data were tested for normality and homoscedasticity, and one-way analysis of variance was performed followed by Duncan's and Tukey's multiple test (*p* < 0.05).

## Results

### Expression and localization of progesterone synthetase and its receptor in sheep follicles

**Figure 1:**
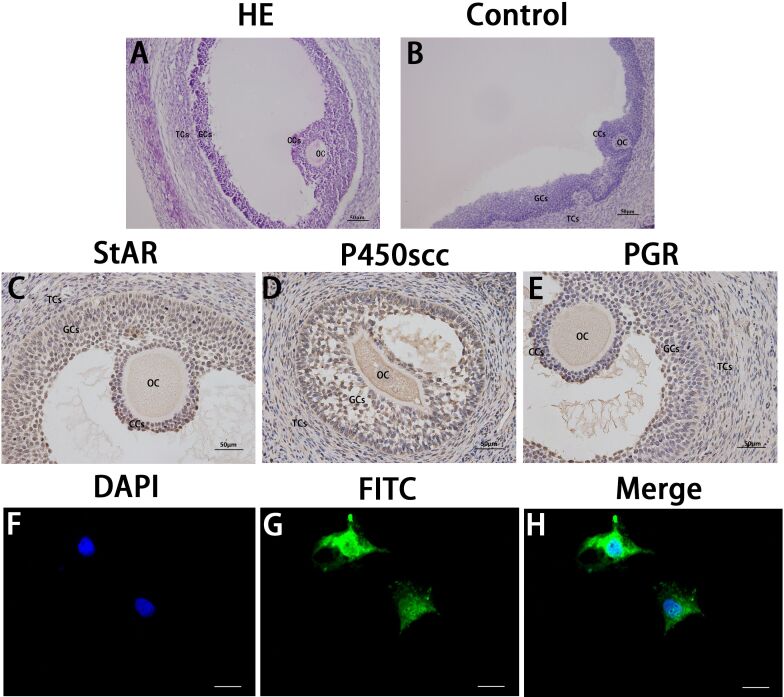
(A) shows the results of Hemathoxylin/Eosin (HE) staining and (B) Negative control. Immunolocalization of StAR (C), P450scc (D) and PGR (E) proteins in sheep follicles; (F-H) shows the immunofluorescence localization of PGR (green) in granulosa cells, and the nuclei are restained with DAPI (blue). Original magnification: Cellular immunofluorescence and immunohistochemistry: x400, HE and negative control: x200. OC: oocyte; CCs: cumulus cells; GCs: granulosa cells; TCs: theca cells.

First, the expression of P4 synthetase and related receptors in sheep follicles was detected with immunohistochemistry. The detection results are shown in Figure 1C-E. The results showing that StAR, P450scc, and PGR were all expressed in sheep GCs. Subsequently, the expression of PGR in GCs was further verified by the cellular immunofluorescence assay.

### P4 content, synthetase and receptor expression at different stages of follicular development in sheep

**Figure 2:**
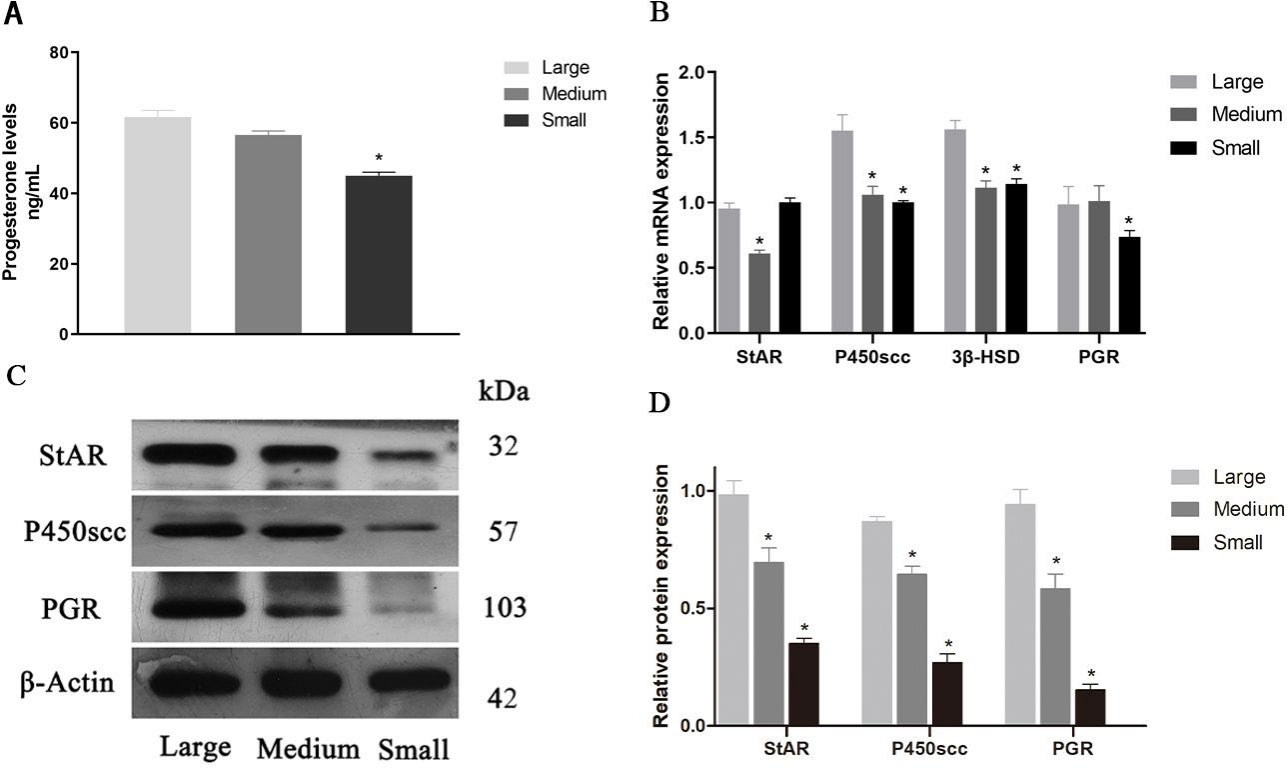
P4 levels, StAR, P450scc, 3β-HSD and PGR levels in different developmental stages of follicles. (A) P4 levels at different follicular development stages; (B) The relative gene levels of StAR, P450scc, 3β-HSD and PGR; (C) Western blot of StAR, P450scc and PGR; (D) the relative protein levels of StAR, P450scc and PGR. β-Actin was used as internal control. Values indicate means ± SEM. *p < 0.05 compared with large follicle group. PGR: Progesterone receptor; SEM: standard error of mean

P4 content and synthetase and receptor expression at different stages of follicular development were detected, as shown in Figure 2. Results showed that the P4 content outcomes in large (n=31), medium (n=44), and small follicles (n=96) detected by ELISA are 61.62 ± 1.91, 56.53 ± 1.17, and 45.05 ± 0.97 ng/mL, respectively.

The results showed that the concentration of P4 in large and medium follicles was significantly higher than that in small follicles (*p* < 0.05). The expression of *StAR* gene in middle follicles was significantly lower than that in large follicles and small follicles, while the expression of *P450scc* and *3β-HSD* gene in middle follicles and small follicles was significantly lower than that in large follicles (*p* < 0.05). In addition, PGR gene expression in small follicles was significantly lower than that in large and medium follicles (Figure 2B). In protein detection, P4 synthetase and receptor PGR in medium and small follicles were significantly lower than those in large follicles (*p* < 0.05) (Figure 2D).

### Effects of FSH on progesterone secretion and receptor expression in GCs

**Figure 3:**
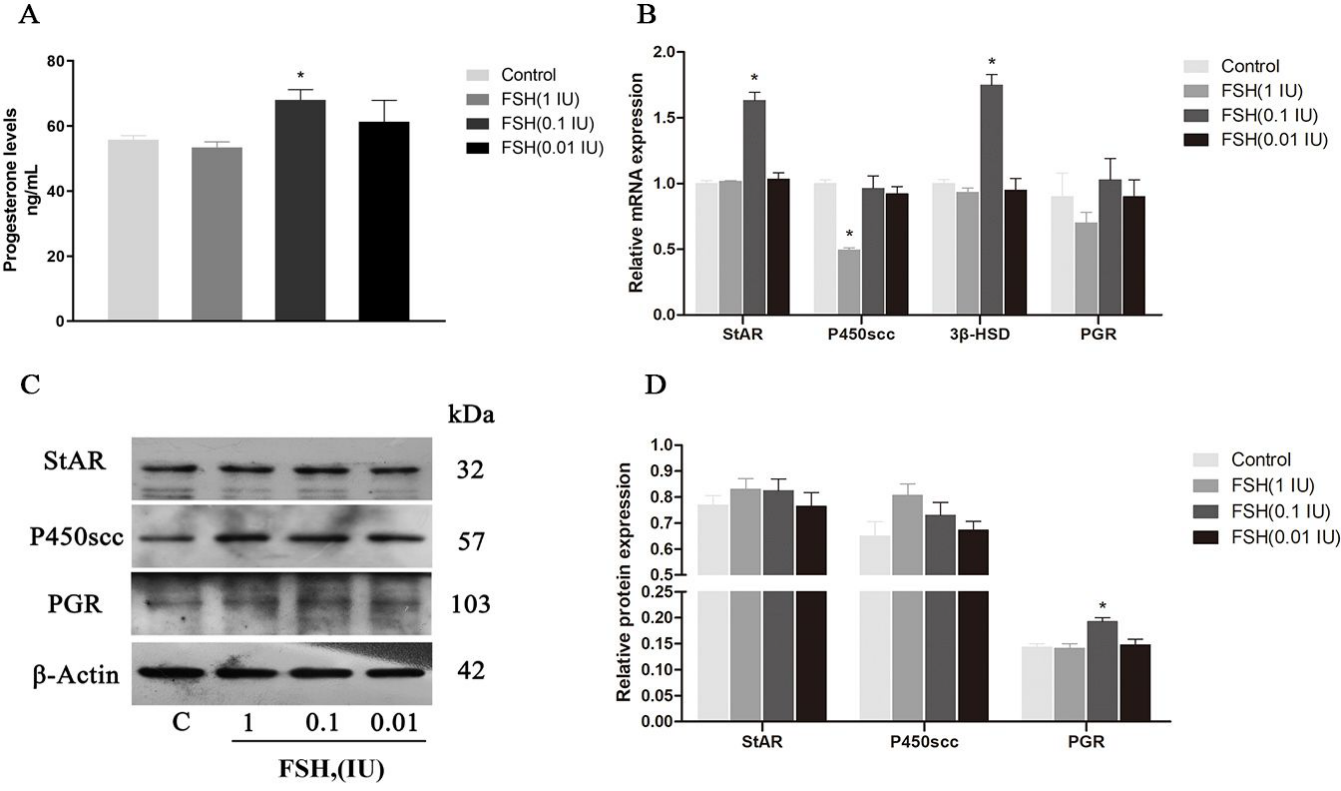
Effect of different concentrations of FSH on P4 secretion (A), gene (B) and protein expression (C, D) of StAR, P450scc, 3β-HSD and PGR in cultured GCs. β-Actin was used as internal control. β-Actin was used as internal control. Values indicate means ± SEM. *p < 0.05 compared with control group. FSH: follicle stimulating hormone; SEM: standard error of mean.

The effects of different concentrations of FSH on P4 content and the expression of related synthetases and receptors are shown in Figure 3. Result showed that the content of P4 after FSH treatment at different concentrations (as detected by ELISA) are 55.83 ± 1.19, 53.46 ± 1.70, 68.04 ± 3.16, and 56.3 ± 1.59 ng/mL, respectively, and the content of P4 in the FSH (0.1 IU) group increases significantly in comparison with the control group (*p* < 0.05). Figure 3B-D shows the gene expressions of StAR, P450scc, 3β-HSD, and PGR, and the protein expressions of StAR, P450scc, and PGR of the different treatment groups. The gene expression of StAR and 3β-HSD in the FSH (0.1 IU) group was significantly higher than that other treated groups and control group (*p* < 0.05). The expression of P450scc in the FSH (1 IU) group was significantly lower than that in the control group (*p* < 0.05). There was no significant difference in PGR gene expression among the treatment and control groups. There was no significant difference in protein expression among the treatment groups with different concentrations of FSH and the control group, although they had a greater expression in relation to the control group. In addition, the relative expression of PGR protein in FSH (0.1 IU) group was significantly higher than that in control group (*p* < 0.05) (Figure 3D).

### Effects of LH on progesterone secretion and receptor expression in GCs

**Figure 4:**
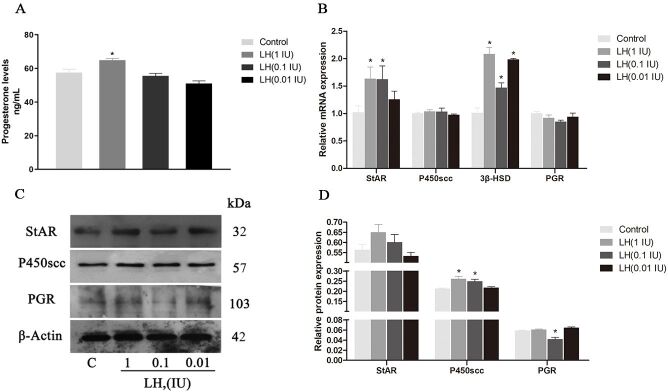
Effect of different concentrations of LH on P4 secretion (A), gene (B) and protein expression (C-D) of StAR, P450scc, 3β-HSD and PGR in cultured GCs. β-Actin was used as internal control. Values indicate means ± SEM. *p < 0.05 compared with control group. LH: luteinizing hormone; SEM: standard error of mean.

The effects of different concentrations of LH on P4 levels, synthetases and receptors are shown in Figure 4. The result showed that P4 levels after treatment with different concentrations of LH by ELISA are 57.50 ±2.00, 64.92 ± 0.91, 55.59 ± 1.52 and 51.08 ± 1.50 ng/mL (n=3).

The results show that the content of P4 in the LH (1 IU) group increased significantly (*p* < 0.05). Additionally, results shows the basal expressions of StAR, P450scc, 3β-HSD, and PGR and the protein expression of StAR, P450scc, and PGR in different treatment groups. The expression of the StAR gene in the LH (1 IU) and LH (0.1 IU) groups increased significantly (*p* < 0.05), and the expressions of 3β-HSD in all treatment groups were significantly higher than that in the control group (*p* < 0.05). There was no significant difference in PGR gene expression between the treatment and control groups. The expression trends of the P450scc gene and protein were the same, but they were higher than that of the control group. However, the histone expressions of LH (1 IU) and LH (0.1 IU) were significantly higher than those of the control group (*p* < 0.05) (Figure 4B-D). The expression levels of the StAR protein were higher than those of the control group, but the difference was not significant. The expression of LH (0.1 IU) in the PGR group was significantly lower than that in the control group (*p* < 0.05).

### Effects of FSH and LH on progesterone secretion and receptor expression in GCs

#### Effects of FSH (1 IU/ml) combined with different concentrations of LH on P4 concentration, synthetase and its receptor expression

**Figure 5:**
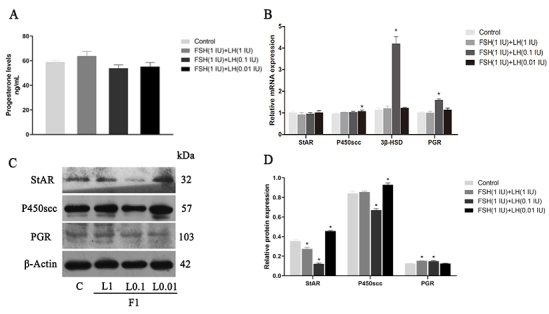
Effects of FSH+LH on P4 secretion and the levels of StAR, P450scc, 3β‐HSD and PGR in cultured GCs. (A) P4 levels; (B) P4 synthetase and receptor relative gene expression; (C, D) Western blotting and relative protein expression of P4 synthetase and receptor. β-Actin was used as internal control. Values indicate means ± SEM. *p < 0.05 compared with control group. F1: FSH (1IU/mL); LH: luteinizing hormone; FSH: follicle stimulating hormone; SEM: standard error of mean.

We further evaluated the effect of FSH and LH combined treatment on P4 in GCs cultured in vitro. Figure 5A shows that the content of P4 detected by ELISA is 59.08 ± 1.19, 63.9 ± 3.62, 54.01 ± 2.72, and 55.32 ± 3.30 ng/mL (n=3), respectively.

The results showed that the content of P4 increased after the FSH 1IU + LH 1 IU (F1 + L1) treatment, but the difference was not significant. Result showed the gene expressions of StAR, P450scc, 3β-HSD, and PGR, and the protein expressions of StAR, P450scc, and PGR in the various treatment groups. Regarding gene expression, P450scc results showed that there was a significant difference between the FSH 1IU + LH 0.1 IU (F1 + L0.01) group and the control group (*p* < 0.05). In 3β-HSD and PGR, the difference between the F1 + L0.1 and the control groups was significant (*p* < 0.05) (Figure 5B). The protein expression results showed that the expressions of StAR in F1 + L1 and F1 + L0.1 were significantly lower than those in the control group (*p* < 0.05), but the expression in the F1 + L0.01 group was significantly higher than that in the control group (*p* < 0.05). The expression of P450scc yielded similar results. F1 + L0.1 was lower than that of the control group (*p* < 0.05), and F1 + L0.01 which was significantly higher than that of the control group (*p* < 0.05) (Figure 5D). The expression of PGR was different from these, and F1 + L1 and F1 + L0.1 were significantly higher than those of the control group (*p* < 0.05).

#### Effects of FSH (0.1 IU/ml) combined with different concentrations of LH on P4 concentration, synthetase and its receptor expression

**Figure 6:**
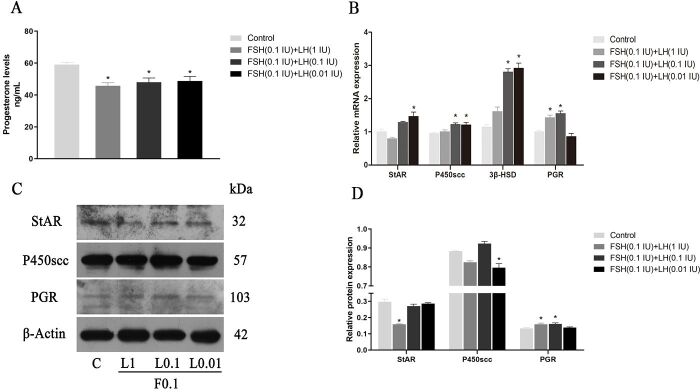
Effects of FSH+LH on P4 secretion and the levels of StAR, P450scc, β‐HSD and PGR in cultured GCs. (A) P4 levels; (B) P4 synthetase and receptor relative gene expression; (C, D) Western blotting and relative protein expression of P4 synthetase and receptor. Values indicate means ± SEM. *p < 0.05 compared with control group. F0.1: FSH (0.1IU/mL); LH: luteinizing hormone; FSH: follicle stimulating hormone; SEM: standard error of mean

Figure 6A respectively shows that the concentrations of P4 (quantified by ELISA) are 59.08 ± 1.19, 45.76 ± 1.9, 47.98 ± 2.65, and 48.77 ± 2.86 ng/mL (n=3).

The results show that the concentrations of P4 are significantly lower in different treatment groups compared with the control group (*p* < 0.05). Figure 6B-D shows the gene expressions of StAR, P450scc, 3β-HSD, and PGR, and the protein expressions of StAR, P450scc, and PGR in different treatment groups. In terms of gene expression, StAR results showed that the expression of the FSH 0.1IU + LH 0.01 IU (F0.1 + L0.01) treatment group was significant higher than that of control group (*p* < 0.05). However, P450scc and 3β-HSD were expressed in the FSH 0.1IU + LH 0.1 IU (F0.1 + L0.1) and F0.1 + L0.01 treatment groups (*p* < 0.05). Protein results showed that the expression of StAR protein in the FSH 0.1IU + LH 1 IU (F0.1 + L1) group was significantly lower than that in the control group (Figure 6D). In P450scc, the F0.1 + L0.01 group was significantly lower than that in the control group (*p* < 0.05). The expression of the PGR protein was consistent with the gene expression, and was significantly higher in the F0.1 + L1 and F0.1 + L0.1 groups compared with the control group (*p* < 0.05).

#### Effects of FSH (0.01 IU/ml) combined with different concentrations of LH on P4 concentration, synthetase and its receptor expression

**Figure 7:**
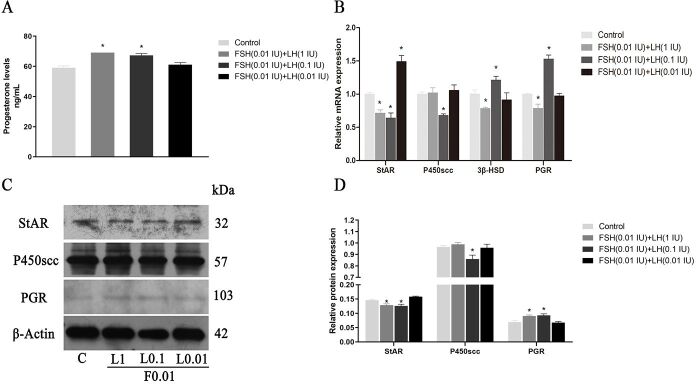
Effects of FSH+LH on P4 secretion and the levels of StAR, P450scc, β‐HSD and PGR in cultured GCs. (A) P4 levels; (B) P4 synthetase and receptor relative gene expression; (C, D) Western blotting and relative protein expression of P4 synthetase and receptor. Values indicate means ± SEM. *p < 0.05 compared with control group. F0.01: FSH (0.01IU/mL); LH: luteinizing hormone; FSH: follicle stimulating hormone; SEM: standard error of mean.

Figure 7A respectively shows that the contents of P4 detected by ELISA are 59.08 ± 1.19, 69.10 ± 1.90, 67.25 ± 1.22, and 61.07 ± 1.4 ng/mL (n=3).

The results show that the expressions of P4 in FSH 0.01IU + LH 1 IU (F0.01 + L1) and FSH 0.01IU + LH 0.1 IU (F0.01 + L0.1) treatment groups increased significantly (*p* < 0.05). Figure 7B-D shows the gene expressions of StAR, P450scc, 3β-HSD, and PGR, and the protein expressions of StAR, P450scc, and PGR in the various treatment groups. The expression of StAR gene in F0.01 + L1 and F0.01 + L0.1 groups were significantly lower than those of the control group, while the expressions of FSH 0.01IU + LH 0.01 IU (F0.01 + L0.01) group were significantly higher than those of the control group. The expressions of P450scc in the F0.01 + L0.1 group were significantly lower than those in the control group. In the results of 3β-HSD and PGR, the expression of the F0.01 + L1 group was significantly lower than that of the control group, while the expression of F0.01 + L0.1 was significantly higher than that of the control group (Figure 7B). In terms of protein expression, StAR results showed that the expressions of F0.01 + L1 and F0.01 + L0.1 groups were significantly lower than those of the control group (*p* < 0.05). The expression of F0.01 + L0.1 in P450scc was significantly lower than that in the control group. The expressions of F0.01 + L1 and F0.01 + L0.1 groups in the PGR were significantly higher than those in the control group.

## Discussion

Granulosa cells can secrete a variety of steroid and cell growth factors and participate in the growth and development of follicles (Wang et al., 2017). In this experiment, we found that StAR and P450scc were expressed in sheep follicular granulosa cells, indicating that granulosa cells can synthesize P4. The expression of StAR, P450scc protein and 3β-HSD mRNA increased gradually with the development of antral follicles. This is consistent with previous studies in humans, cattle and mice (Erickson et al., 1991; Garverick et al., 2002; Ji et al., 2020). This suggests that there may be differences in the ability of granulosa cells to synthesize P4 during the development of antral follicles in sheep. In order to test this hypothesis, the concentration of P4 in follicular fluid of different sizes was detected. The results showed that the concentration of P4 in large and medium follicles was significantly higher than that in small follicles. During the development of follicles, the increase in steroid synthesis and the level of granulosa cell luteinization may be two of the main reasons responsible for the increases of GCs P4 synthetase and the concentration of P4 in follicular fluid. However, the lowest abundance of StAR was found in large follicles of cattle (Soumano and Price, 1997). This may be attributed to differences between species or the effects of postovulation detection, thus suggesting that P4 has different regulatory effects on follicles of different sizes. P4 can play a biological role through its specific receptor PGR (Mishra et al., 2015). The PGR expression was found in both follicular tissue and cultured GCs in vitro, thus indicating that GCs are also one of the target cells of P4. Following the development of follicles, the expression of PGR protein increased, but the expression of its mRNA did not change significantly (medium). Differences in gene and protein expression may be attributed to temporal and spatial differences in gene transcription and protein translation (Sousa Abreu et al., 2009). However, these results suggest that autocrine P4 in follicles is mediated by PGR receptors on GCs during follicular development in sheep.

FSH is involved in the development of antral follicles (Sekar et al., 2000b; Shih et al., 2011). In this experiment, different concentrations of FSH up-regulated progesterone secretion and synthetase expression in granulosa cells in vitro. These findings are similar with the results obtained from pigs (Lin and Rui, 2010). These results indicate that FSH regulates the expression of StAR and 3β-HSD genes, affects P4 synthesis, and regulates GCs function. However, P450scc was significantly reduced in FSH (1 IU) group. This is consistent with previous studies in pigs, in which P450scc expression was up-regulated at low concentrations and down-regulated at high concentrations of FSH (Yang et al., 2001). This may have resulted in a lower P4 concentration at 1 IU rather than at 0.1 IU. In addition, in the detection of PGR, 0.1 IU FSH significantly up-regulated the expression of PGR. FSH was shown to promote the expression of PGR in mouse GCs (Park-Sarge and Mayo, 1994). Nevertheless, PGR expression in porcine cumulus cells treated with FSH + E2 + P4 and FSH + E2 in vitro was higher than that in the control group (Kawashima et al., 2008). Increased expression of these PGR may be associated with increased P4 synthesis.

LH also regulates follicular development. We observed that LH doses of 1 and 0.1 IU up-regulated the gene and protein expressions of StAR, and also up-regulated the protein expression of P450scc, but the gene expression levels were not significant. The effects of LH on 3β-HSD were promotion is significant. Studies in mice and llamas have shown that LH can up-regulate the mRNA expression of StAR and 3β-HSD (Valderrama et al., 2020; Kundu et al., 2012). This was consistent with our experimental results. This shows LH is also involved in the synthesis of P4 in GCs the same time. LH can stimulate the expression of the luteinizing hormone receptor (LHR) on granulocytes, thereby enabling LH to exert its biological function to a greater extent, which may account for the increased P4 synthesis (Mihm et al., 2006; Lydon et al., 1995). In addition, we found that protein expression results of PGR were reduced at 0.1 IU, which was speculated to be related to the temporary promotion of PGR mRNA expression of LH. This may be related to LH secretion fluctuations and follicular cycle. Following the proliferation of LH in GCs, increased P4 synthesis and rapid induction of PGR are all key regulatory mechanisms that occur during the ovulation cycle (Hughes and Murphy, 2021).

The development of follicles depends on the synergistic action of FSH and LH. After treatment with different concentrations of FSH and LH, we found that LH had no effect on the secretion of P4 by follicular GCs at high concentrations of FSH. When FSH is at medium concentration, LH down-regulates the secretion of P4. When the FSH concentration is low, LH promote progesterone secretion, and in the early stage of follicular development, FSH induces the expression of LHR. Subsequently, the concentration of LH increases, which in turn increases E2 synthesis in GCs and weakens their ability to secrete P4 (Lydon et al., 1995). However, in the late stage of follicular development, the concentration of FSH decreases significantly, and LH promotes the secretion of P4 by GCs and induces ovulation (Mihm et al., 2006). These results suggest that the concentrations of FSH and LH change dynamically at different stages of follicular development. In this study, the co-treatment of FSH and LH showed that the expression of StAR decreased with the increase of LH, which was contrary to the experimental results of this experiment earlier adding LH alone. Furthermore, FSH down-regulated the expression of StAR in bovine follicular GCs, but increased the concentration of P4 (Zheng et al., 2008). However, there was no difference in StAR at 24 h after HCG treatment in macaque granulocytes (Chaffin et al., 2000). These different effects on StAR may be related to species and treatment methods. In addition, FSH concentration significantly up-regulated the expression of the 3β-HSD gene when medium concentration with LH was added. In addition, the expression of 3β-HSD was significantly up-regulated by FSH (0.1 IU), and even at low LH concentrations, FSH could significantly up-regulate the expression of 3β-HSD. This result was similar to that of FSH and LH alone in promoting 3β-HSD expression. And in the FSH0.1 group, changes in P4 concentration were consistent with related synthetase trends. Therefore, we believe that FSH and LH play a synergistic role in regulating the secretion of P4 by follicular GCs. In addition, LH (1 IU) and 0.1 IU significantly up-regulated the protein and mRNA expressions of PGR in different concentrations of FSH groups. PGR was significantly increased in porcine cumulus cells treated with hCG (Kawashima et al., 2008), PGR was significantly up-regulated in cumulus cells treated with FSH and LH (0.05 IU) (Blaschka et al., 2019). This is similar with our experimental results, thus indicating that the expression of PGR requires the synergistic effects of FSH and LH, and LH plays a dominant role in its regulation.

## Conclusion

P4 synthetase and PGR were differentially expressed during follicular development, and P4 synthesis was regulated in GCs cultured in vitro after treatment with FSH and LH. This suggested that FSH and LH are involved in follicular development through the regulation of P4 secretion and PGR expression in GCs. PGR expression was more dependent on LH action, whereas FSH reduced such expression. Overall, this provided additional information regarding the regulation of steroid hormone synthesis and follicle development in sheep.
